# Genomic Analysis of Antibiotic-Resistant *Staphylococcus epidermidis* Isolates From Clinical Sources in the Kwazulu-Natal Province, South Africa

**DOI:** 10.3389/fmicb.2021.656306

**Published:** 2021-08-05

**Authors:** Jonathan Asante, Bakoena A. Hetsa, Daniel G. Amoako, Akebe L. K. Abia, Linda A. Bester, Sabiha Y. Essack

**Affiliations:** ^1^Antimicrobial Research Unit, College of Health Sciences, University of KwaZulu-Natal, Durban, South Africa; ^2^School of Laboratory Medicine and Medical Sciences, University of KwaZulu-Natal, Durban, South Africa; ^3^Biomedical Research Unit, University of KwaZulu-Natal, Durban, South Africa

**Keywords:** *Staphylococcus epidermidis*, antibiotic resistance, whole-genome sequencing, genomics, coagulase-negative, staphylococci

## Abstract

*Staphylococcus epidermidis* has become an important nosocomial pathogen. Multidrug resistance makes *S. epidermidis* infections difficult to treat. The study aims to describe the genomic characteristics of methicillin-resistant *S. epidermidis* (MRSE) isolated from clinical sources, to comprehend the genetic basis of antibiotic resistance, virulence, and potential pathogenicity. Sixteen MRSE underwent whole-genome sequencing, and bioinformatics analyses were carried out to ascertain their resistome, virulome, mobilome, clonality, and phylogenomic relationships. In all, 75% of isolates displayed multidrug resistance and were associated with the carriage of multiple resistance genes including *mecA*, *blaZ*, *tet(K)*, *erm(A)*, *erm(B)*, *erm(C)*, *dfrG*, *aac(6′)-aph(2′′)*, and *cat(pC221)* conferring resistance to β-lactams, tetracyclines, macrolide–lincosamide–streptogramin B, aminoglycosides, and phenicols, which were located on both plasmids and chromosomes. Their virulence profiles were evidenced by the presence of genes involved in adherence/biofilm formation (*icaA*, *icaB*, *icaC*, *atl*, *ebh*, and *ebp*), immune evasion (*adsA*, *capC*, and *manA*), and antiphagocytosis (*rmlC*, *cdsA*, and *A*). The community-acquired SCC*mec* type IV was the most common SCC*mec* type. The CoNS belonged to seven multilocus sequence types (MLSTs) and carried a diversity of mobile genetic elements such as phages, insertion sequences, and plasmids. The bacterial anti-phage defense systems clustered regularly interspaced short palindromic repeats/CRISPR-associated (CRISPR-Cas) immunity phage system and restriction-modification system (R-M system) and the arginine catabolic mobile element (ACME) involved in immune evasion and transport of virulence genes were also found. The insertion sequence, IS256, linked with virulence, was found in 56.3% of isolates. Generally, the isolates clustered according to STs, with some similarity but also considerable variability within isolates. Whole-genome sequencing and bioinformatics analysis provide insights into the likely pathogenicity and antibiotic resistance of *S. epidermidis*, necessitating surveillance of this emerging pathogen.

## Introduction

*Staphylococcus epidermidis* are coagulase-negative staphylococci (CoNS) that are commensals of the skin microbiome. Among the CoNS, *S. epidermidis* and *Staphylococcus haemolyticus*, together referred to as the *S. epidermidis* group, are the most prevalent in clinical settings (Azih and Enabulele, 2013) and can behave as pathogens by colonizing medical devices, infecting surgical wounds, and causing bacteremia (Cabrera-Contreras et al., 2019). CoNS infection is commonly associated with device-associated healthcare infection. *S. epidermidis* is considered clinically relevant, moderately pathogenic, and known to display multidrug and methicillin resistance that complicates treatment (Becker et al., 2014; Xu et al., 2015). *mecA* gene, which mediates methicillin resistance, is highly conserved in *S. epidermidis*, easily transferred to other staphylococcal species by horizontal transmission (Méric et al., 2015), and borne on the staphylococcal cassette chromosome, SCC*mec*, which is shared between *S. epidermidis* and *Staphylococcus aureus* (Méric et al., 2015). It is estimated that globally, *S. epidermidis* together with other CoNS and *S. aureus* causes 30% of hospital-associated infections (Xu et al., 2018).

*Staphylococcus epidermidis* is an important carrier of antibiotic resistance genes (ARGs), which can be transferred between staphylococcal species (Xu et al., 2018). The pathogenicity of *S. epidermidis* is further enhanced by virulence genes associated with adherence/biofilm formation, phenol-soluble modulins, and various mobile genetic elements (MGEs) such as plasmids, insertion sequences (ISs), transposons, pathogenicity islands, and phages that are involved in the acquisition and transmission of resistance and virulence characteristics (Bouchami et al., 2016; Rolo et al., 2017). The arginine catabolic mobile element (ACME) system, a pathogenicity island thought to facilitate host colonization and immune evasion, has generated interest in recent years (O’Connor et al., 2018). According to recent phylogenetic studies, the ACME most likely originated from *S. epidermidis* and transmitted to *S. aureus* through horizontal transfer (Onishi et al., 2013; Planet et al., 2013). Other factors that have been associated with pathogenicity in *S. epidermidis* include the metabolic state of the bacterial cell, genomic rearrangements in pathogenic isolates facilitated by IS256, and the conjugative transfer of antibiotic resistance (Cabrera-Contreras et al., 2019).

Analyses of *S. epidermidis* from diverse geographic locations and clinical origins have shown that the most common STs were ST2, ST59, and ST23, with 74% of screened isolates belonging to the clonal complex 2 (Miragaia et al., 2007). The sequence type ST2, a single *S. epidermidis* lineage, is dominant in hospital environments (Lee et al., 2018). The widespread sequence types ST5, ST12, and ST23 have been reported to exhibit high resistance against most antibiotic drug classes (Martínez-Meléndez et al., 2016). Additionally, there is increasing rifampicin resistance in *S. epidermidis* isolates belonging to ST2 and ST23 in Europe, the United States, and Australia (Lee et al., 2018). This observed resistance conferred by mutations in *rpoB* gene has independently emerged, supporting the assertion that few, well-adapted clonal lineages of *S. epidermidis* are abound in clinical environments (Lee et al., 2018).

In South Africa, a study on catheter-related bloodstream infections (CRBSIs) in a hospital in Pretoria, identified *S. epidermidis* as the causative organism in 31% of CRBSI cases (Ehlers et al., 2018). All the *S. epidermidis* isolates identified were resistant to β-lactams, all carried *mecA* gene (100%), and 83% carried the IS element IS256. There is however a paucity of studies characterizing the virulence and resistance mechanisms of *S. epidermidis* in South Africa and Africa more generally.

In this study, we describe the genomic characteristics of methicillin-resistant *S. epidermidis* (MRSE) isolated from hospitals within the KwaZulu-Natal province in South Africa, specifically their resistome, virulome, mobilome, clonality, and phylogenies together with associations between them and other parameters to gain insights into the genetic basis of antibiotic resistance, virulence, and potential pathogenicity.

## Materials and Methods

### Description of Strains and Antimicrobial Susceptibility Testing

Sixteen MRSE isolates, selected as a subsample from a previous study (Asante et al., 2021) based on their resistance profiles, were subjected to whole-genome sequencing (WGS). Briefly, an initial 89 suspected CoNS isolates were collected from blood cultures sourced from hospitals within the uMgungundlovu District in the KwaZulu-Natal Province. Isolates were selected based on their resistance to cefoxitin and to multiple antibiotics. MRSE isolates were selected based on their well-documented role as a frequent cause of nosocomial infection and ability to form biofilms (Becker et al., 2014). The 16 clinical MRSE isolates collected from patients in three hospitals in the uMgungundlovu District in the KwaZulu-Natal Province, South Africa, had been subjected to initial identification by Gram staining, colony characteristics, and the Staphaurex^TM^ Latex Agglutination Test (Thermo Scientific, Kent, United Kingdom). Speciation of isolates was done using the automated VITEK 2 system (BioMérieux, Marcy-L’Etoile, France).

The antibiotypes for 20 antibiotics were determined by the Kirby–Bauer disk-diffusion method or by the broth microdilution method. The Clinical and Laboratory Standards Institute (CLSI) guidelines were used to interpret their antimicrobial susceptibility (Clinical and Laboratory Standards Institute, 2016). Antibiotics tested (disk diffusion) included penicillin G (10 μg), cefoxitin (30 μg), ceftaroline (30 μg), ciprofloxacin (5 μg), moxifloxacin (5 μg), azithromycin (15 μg), erythromycin (15 μg), gentamicin (120 μg), amikacin (30 μg), chloramphenicol (30 μg), tetracycline (30 μg), doxycycline (30 μg), tigecycline (15 μg), teicoplanin (30 μg), linezolid (30 μg), clindamycin (10 μg), rifampicin (5 μg), sulfamethoxazole/trimethoprim (1.25/23.75 μg), and nitrofurantoin (300 μg). The minimum inhibitory concentration (MIC) of vancomycin was determined by the broth microdilution method according to CLSI guidelines (Clinical and Laboratory Standards Institute, 2016). The cefoxitin disk diffusion was used to detect methicillin resistance and molecularly confirmed by PCR detection of *mecA* gene.

### Phenotypic Detection of Biofilm Formation

The biofilm-forming abilities of isolates were assessed by the tissue culture plate method described by Mitchell et al. (2010). Briefly, isolates were grown in trypticase soy broth (TSB) supplemented with glucose for 24 h at 37°C. Sterile 96-well microtiter plates were inoculated with bacterial suspension adjusted to 0.5 MacFarland standard and incubated at 37°C for 24 h. The plates were washed after incubation and dried at room temperature. The wells then stained with 0.1% crystal violet solution, incubated at room temperature for 10 min, and washed thrice with distilled water. A 30% acetic acid solution was added to solubilize the crystal violet retained by the biofilm. The optical densities of samples in each well were read at 570 nm using a microtiter plate reader (BMG LABTECH, Offenburg, Germany). *S. epidermidis* ATCC 35984 was used as positive control. Isolates were categorized as strong, moderate, weak, and non-biofilm formers, using the formula OD < ODC = no biofilm producer, ODC < OD ≤ (2 × ODC) = weak biofilm producer, (2ODC) < OD ≤ (4 × ODC) = moderate biofilm producer, and (4 × ODC) < OD = strong biofilm producer, where ODC is the average OD of the negative control.

### Whole-Genome Sequencing

Genomic DNA (gDNA) from pure colonies of MRSE isolates grown from overnight cultures was extracted and purified using the GenElute Bacterial Genomic DNA kit (Sigma Aldrich, St. Louis, MO, United States), according to the manufacturer’s instructions. DNA was checked by agarose gel electrophoresis, while the concentration and purity were determined using Nanodrop^TM^ 1000 spectrophotometer (Thermo Scientific, Wilmington, DE, United States). For library preparation, the Nextera XT DNA Sample Preparation Kit was used to generate paired-end libraries, followed by WGS on the Illumina MiSeq platform (Illumina, San Diego, CA, United States) at the Sequencing Core Facility, National Institute for Communicable Diseases, Johannesburg, South Africa.

Quality trimming of the sequence reads was done by the use of Sickle version 1.33^1^, while assembler, SPAdes version 3.11 (Bankevich et al., 2012), and the CLC Genomics Workbench version 10.1 (CLC, Bio-QIAGEN, Aarhus, Denmark) were used for *de novo* assembly of the reads. The assembled contiguous sequences were submitted via the National Center for Biotechnology Information (NCBI) Prokaryotic Genome Annotation Pipeline to GenBank for gene annotation. The generated contigs were analyzed further to investigate genetic elements of interest.

### Bioinformatics Analyses

#### Pathogenicity, Single-Nucleotide Polymorphism Calling, Resistome, and Virulome Analyses

The identities of isolates and observed phenotypic resistance were confirmed by the genomic data using the Pathogenwatch platform^2^. The prediction of isolates’ pathogenicity toward human hosts was determined by PathogenFinder available at https://cge.cbs.dtu.dk/services/PathogenFinder/. The assembled genomes from the WGS data were annotated to predict and identify the resistome using ResFinder 4.1 (with a minimum length and threshold of 60% and 90%, respectively), and the Comprehensive Antibiotic Resistance Database (CARD)^3^ (Alcock et al., 2020), using the default selection criteria “perfect and strict hits only.” The platforms were used side by side to compensate for the inherent deficits in individual platforms.

We determined the genetic basis (chromosomal single-nucleotide polymorphism [SNP]) for observed fluoroquinolone and rifampicin resistance from the assembled genomes by investigating mutations conferring resistance to fluoroquinolones and rifampicin using BLASTn (Altschul et al., 1990). Briefly, *gyrA*, *gyrB*, *parC*, *parE*, and *rpoB* genes in a reference susceptible *S. epidermidis* (*S. epidermidis* strain ATCC 12228) were aligned with the corresponding genes from resistant isolates in this study with BLASTn to call for SNPs in those genes using the Clustal Omega tool (European Molecular Biology Laboratory). Thus, the mutations in the genomes of the study isolates were manually curated.

VirulenceFinder 2.0 (using a minimum length of 60% and a threshold of 90%)^4^ (Joensen et al., 2014), virulence factor database (VFDB)^5^, and BacWGSTdb^6^ were used to screen for the presence of virulence genes. Various virulence determinants consistent with different major virulence factors (including adherence, enzymes, immune evasion, secretion system, toxins, anti-phagocytosis, biofilm formation/adherence, and intracellular survival) associated with *S. epidermidis* were investigated.

#### *In silico* Multilocus Sequence Typing

Multilocus sequence typing (MLST) was performed *in silico* using MLST 2.0 program software^7^ available on the website of the Center for Genomic Epidemiology (Larsen et al., 2012) and the public molecular typing database, PubMLST^8^. Sequence types were assigned by matching the internal fragments of the seven housekeeping genes (*arcC*, *aroE*, *gtr*, *mutS*, *pyrR*, *tpiA*, and *yqiL*) from *S. epidermidis* to identify alleles (Thomas et al., 2007). We performed eBURST analyses (Feil et al., 2004) in the MLST database to identify clones similar to obtained STs.

#### Identification of Mobile Genetic Elements and Genetic Support Environment

Mobile genetic elements associated with ARGs and their genetic context were investigated using NCBI annotations. RAST 2.0 (Aziz et al., 2008) was used to ascertain MGEs and the genetic support environment. The web-based typing tool SCC*mec*Finder^9^ was used for the *in silico* determination of the SCC*mec* types and their structural position in the MRSE isolates. *In silico* detection of plasmid replicon types was done out using PlasmidFinder 2.1, available at https://cge.cbs.dtu.dk/services/PlasmidFinder/ (Carattoli et al., 2014).

The PHASTER tool^10^ was used to identify and annotate prophage sequences within the genomes (Arndt et al., 2016). Only the prophage regions identified as “intact” by PHASTER were considered. The region positions of the prophages were BLASTED on CARD to determine if the prophages harbored resistance genes. ISs and transposons flanking the resistance genes were identified using the MobileElementFinder v1.0.3 (2020-10-09)^11^ (Johansson et al., 2020), available on the website of the Center for Genomic Epidemiology^12^. We used NCBI annotations to determine the support environment of the resistance genes.

#### Clustered Regularly Interspaced Short Palindromic Repeats/CRISPR-Associated (CRISPR-Cas System), Arginine Catabolic Mobile Element, and Restriction-Modification System

We searched for clustered regularly interspaced short palindromic repeats (CRISPR) and cas genes in the sequence data using the CRISPRCasFinder, available at https://crisprcas.i2bc.paris-saclay.fr/CrisprCasFinder/Index, using the default advanced settings for CRISPR and the clustering model “SubTyping” for Cas. Restriction-ModificationFinder 1.1, available at https://cge.cbs.dtu.dk/services/Restriction-ModificationFinder/, was used to investigate the presence of the restriction-modification system (R-M system), using a minimum length of 60% and a threshold for%ID of 95% (Roer et al., 2016). The ACME genes within the genomes were detected and aligned. Alignment of the ACME components made up of the *arc* operon, the *opp3* operon, and the *kdp* operon, which was used to classify the ACME components as follows: *arc* and *opp3* operons (type I), the *arc* operon only (type II), the *opp3* operon only (type III), the *arc* and *kdp* operons (type IV), and all three *arc*, *opp*, and *kdp* operons (type V), using Pathosystems Resource Integration Center (PATRIC)^13^ annotations.

#### Phylogenetic Analyses Using Whole-Genome Sequencing Single-Nucleotide Polymorphisms and Whole-Genome Sequencing Multilocus Sequence Type Trees

Phylogenetic trees were constructed based on the maximum likelihood method using the CSIPhylogeny^14^ (Kaas et al., 2014), which performs SNP calling, filtering of the SNPs, and inferring phylogeny based on the concatenated alignment of the high-quality SNPs, using the assembled contigs. The analysis was performed on the platform using default parameters, as follows: minimum depth at SNP positions of 10×; minimum relative depth at SNP positions of 10%; minimum distance between SNPs (prune) at 10 bp; minimum SNP quality of 30; minimum read mapping quality of 25; and minimum Z-score of 1.96. *S. aureus* ATCC 25923 was used to root the tree, facilitating the configuration of the phylogenetic distance between the isolates on the branches. Assembled genomes for comparison were uploaded. To see how our isolates compare with *S. epidermidis* genomes from Africa, we searched and downloaded *S. epidermidis* genomes reported in Africa and curated on the PATRIC website and included them in the analysis. To edit and visualize the phylogenetic tree, we used the Figtree program^15^. We used Phandango (Hadfield et al., 2018) to visualize the phylogeny in association with the isolate demographics, resistance mechanisms, and *in silico* WGS typing metadata.

### Nucleotide Sequence Accession Numbers

The nucleotide sequences of the 16 MRSE strains (C35, C36, C38, C40, C68, C81, C119, C122, C127, C133, C135, C137, C138, C145, C146, and C148) used in this work were uploaded in GenBank database in the Bioproject number PRJNA667485.

## Results

### Phenotypic Characteristics of Methicillin-Resistant *Staphylococcus epidermidis* Isolates

Sixteen blood culture MRSE isolates were used in the WGS analysis. Methicillin resistance was confirmed by PCR detection of *mecA* gene. Twelve (75%) were multidrug-resistant, defined as resistance to at least one antibiotic in three or more distinct antibiotic classes (Magiorakos et al., 2012). Two isolates were resistant to the anti-MRSE cephalosporin ceftaroline, while one and two isolates were resistant to tigecycline and teicoplanin, respectively. None of the isolates were resistant to vancomycin; however, one isolate showed intermediate susceptibility to vancomycin, while one isolate each was resistant to linezolid and nitrofurantoin. Antibiotic susceptibility profiles of MRSE isolates used in this study are shown in Table 1.

**TABLE 1:** Antibiotic susceptibility and demographic characteristics of the MRSE collected from blood cultures.

Isolate ID	Sex	Ward	Antibiotic resistance profile																			
			FOX	PEN	CPT	CIP	MXF	AZM	ERY	GEN	AMK	CHL	TET	DOX	TGC	TEC	LZD	CLI	RIF	SXT	NIT	VAN
C35	F	E1 Pediatric ward	R	R	R	R	R	R	R	S	S	R	R	R	S	S	S	R	S	R	S	S
C36	M	Neonatal ICU	R	R	S	R	I	R	R	S	I	R	R	R	R	R	R	I	R	R	R	S
C38	F	H2 Medical ward	R	R	S	R	R	R	R	S	S	S	S	S	S	S	S	S	S	R	S	S
C40	M	3N Extension ward	R	R	S	S	R	R	R	S	S	S	S	S	S	S	S	S	S	R	S	S
C68	M	7F Pediatric ward	R	R	S	I	S	R	R	S	S	R	R	R	S	S	S	S	S	R	S	S
C81	F	F2 Surgical ward	R	R	S	R	R	R	R	S	S	S	S	S	S	S	S	R	R	R	S	S
C119	M	2F Pediatric ICU	R	R	R	S	S	R	R	S	S	S	R	R	S	I	S	S	S	R	S	I
C122	M	Pediatric OPD	R	R	S	S	S	S	S	S	S	S	S	S	S	S	S	S	S	R	S	S
C127	M	1F Male surgical ward	R	R	S	S	S	R	R	S	S	S	S	S	S	S	S	S	S	R	S	S
C133	M	Pediatric OPD	R	R	S	S	S	S	S	S	S	S	S	S	S	S	S	S	S	R	S	S
C135	M	Pediatric OPD	R	R	S	R	R	R	R	S	S	R	R	R	S	S	S	S	S	R	S	S
C137	U	Ward O	R	R	S	R	I	R	R	I	S	S	S	S	S	S	S	R	S	R	S	S
C138	F	H Ward	R	R	S	S	S	S	S	S	S	S	S	S	S	S	S	S	S	R	S	S
C145	M	Casualty	R	R	S	S	S	R	R	S	S	S	S	S	S	R	S	S	S	I	S	S
C146	M	Pediatric ward	R	R	S	S	S	S	S	S	S	S	S	S	S	S	S	S	R	R	S	S
C148	F	D5 Ward	R	R	S	S	S	R	R	S	S	S	S	S	S	S	S	S	S	S	S	S

*Antibiotic susceptibility tests were interpreted according to the CLSI breakpoints for coagulase-negative staphylococci. FOX, cefoxitin; PEN, penicillin; CPT, ceftaroline; CIP, ciprofloxacin; MXF, moxifloxacin; AZM, azithromycin; ERY, erythromycin; GEN, gentamicin; AMK, amikacin; CHL, chloramphenicol; TET, tetracycline; DOX, doxycycline; TGC, tigecycline; TEC, teicoplanin; LZD, linezolid; CLI, clindamycin; RIF, rifampicin; SXT, sulfamethoxazole/trimethoprim; NIT, nitrofurantoin; VAN, vancomycin; R, resistant; I, intermediate; S, susceptible; M, male; F, female; U, unknown; OPD, outpatient department; ICU, intensive care unit; +, present; −, absent; M, male; F, female; U, unknown; MRSE, methicillin-resistant *Staphylococcus epidermidis*.*

### Genome and Assembly Features, and Characterization of Resistome

The genome and assembly characteristics of the sequences, including size, number of contigs, number of RNAs, guanine–cytosine (GC) content (%), number of coding sequences, N_50_, and L_50_ are shown in Table 2. The isolates’ draft genome size ranged from 1.9 Mb to 2.9 Mb, with a GC content of 31.7% to 32.5%. ARGs conferring resistance to β-lactams (*mecA* and *blaZ*), tetracyclines [*tet(K)* and *tet(M)*], macrolide–lincosamide–streptogramin B antibiotic (MLS_B_) [*erm(A)*, *erm(B)*, *erm(C)*, *msr(A)*, and *mph(C)*], trimethoprim-sulfamethoxazole (*dfrG*), aminoglycosides [*aac(6′)-aph(2″)*, *aph(3′)-III*, and *aadD*], chloramphenicol [*cat(pC221)* and *cat(pC233)*], and fosfomycin [*fosB*] were detected in isolates (Table 3). All MRSE isolates possessed either *mecA* or *blaZ* gene. Genetic resistance determinants for tigecycline-, teicoplanin-, linezolid-, and nitrofurantoin-resistant phenotypes are currently under investigation (He et al., 2019; Wardenburg et al., 2019; Yushchuk et al., 2020). There was discordance between resistance phenotype and ARGs, relating to trimethoprim- sulfamethoxazole-, tetracycline-, doxycycline-, rifampicin-, and erythromycin-resistant phenotypes. Even though some isolates were phenotypically resistant to these antibiotics, no corresponding ARGs were detected.

**TABLE 2:** Genome and assembly characteristics of sequenced MRSE isolates from clinical sources.

Isolate ID	Size (Mb)	GC%	Contigs	No. of RNAs	No. of coding sequences	*N*_50_	*L*_50_
C35	2.9	31.8	552	15	3,185	6,031	166
C36	2.8	32.0	177	75	53,309	53,309	17
C38	2.7	32.4	61	82	2,657	91,621	10
C40	2.5	31.8	53	63	2,487	94,191	9
C68	2.8	31.9	140	65	2,834	82,977	10
C81	2.8	31.9	268	68	2,959	25,474	37
C119	2.4	32.5	1,331	34	3,385	3,259	146
C122	2.7	31.7	1,267	45	3,609	9,443	65
C127	2.8	31.8	255	92	2,922	31,786	27
C133	2.4	32.0	22	62	2,295	348,106	3
C135	2.8	32.2	678	65	3,171	9,750	72
C137	1.9	32.1	141	61	1,987	24,369	23
C138	2.9	31.9	541	63	3,156	20,755	35
C145	2.6	32.2	103	90	2,597	126,613	7
C146	2.3	32.0	25	56	2,188	171,915	4
C148	2.5	32.5	117	68	2,587	315,043	3

*N_50_, smallest contig of the size-sorted contigs that make up at least 50% of the respective assembly. L_50_, number of contigs that make up at least 50% of the respective total assembly length. MRSE, methicillin-resistant *Staphylococcus epidermidis*; GC, guanine–cytosine.*

**TABLE 3:** Genotypic characteristics of the MRSE isolates.

Isolate	Resistome (plasmid/chromosomal-mediated)	Plasmid replicon type	R-M system	*SCC*mec* type	ACME type	MLST	Insertion sequences	No. of CRISPR-Cas elements	Pathogenicity score (no. of pathogenic families)
C35	*mecA*, *blaZ*, *norA*, *dfrG*, *tet(K)*, *cat(pC221)*, *erm(C)*	rep15, rep19b, rep19c, rep24c, rep39, rep21, repUS22, rep7a, repUS43, rep10	–	SCC*mec* type IV(2B)	–	Unknown^#^	IS256, ISSau4, ISSep3	10 (0)	0.944 (482)
C36	*MecA*, *blaZ*, *tet(M)*, *aac(6′)-aph(2′′)*, *aadD*, *cat(pC221)*, *cat(pC233)*, *erm(A)*, *erm(C)*, *norA*	rep19b, repUS9, rep10, rep22, rep7a, rep7b, repUS43	Type II	SCCmec type V(5C2)	–	ST54	IS256	5 (0)	0.942 (540)
C38	*MecA*, *blaZ*, *norA*, *erm(C)*	rep10, repUS43	Type II	SCC*mec* type IV(2B)	–	ST83	ISSep3, ISSau4, IS256	3 (0)	0.943 (555)
C40	*MecA*, *blaZ*, *aac(6′)-aph(2′′)*, *erm(A)*, *erm(C)*	rep10, repUS9, repUS43	–	–	–	ST54	IS256	4 (0)	0.944 (521)
C68	*MecA*, *blaZ*, *tet(M)*, *tet(K)*, *cat(pC221)*, *erm(C)*, *lsa(A)*, *erm(B)*, *dfrG*	rep2, repUS11, rep10, rep7a, repUS43, repUS12	–	SCC*mec* type XIII(9A)	III	ST210	ISSau4, ISEfa11	6 (0)	0.947 (501)
C81	*MecA*, *blaZ*, *norA*, *aac(6′)-aph(2′′)*, *cat(pC221)*, *erm(A)*, *erm(C)*, *erm(B)*	rep7a, repUS43, rep2, repUS11, rep22, repUS46, repUS23, repUS9, rep10	–	SCC*mec* type IV(2B)	–	ST2	ISEfa11, ISSep3, ISSau4, IS256	5 (0)	0.942 (531)
C119	*mecA*, *blaZ*, *erm(C)*, *aac(6′)-aph(2′′)*, *aac(6′)-Ic*, *dfrG*	rep10, rep19b, rep19c, rep20, rep39, repUS70	–	–	III	Unknown	IS16, ISSep2, IS256	6 (0)	0.947 (171)
C122	*mecA*, *blaZ*, *aadD*, *aac(6′)-aph(2′′)*, *mph(C)*, *msr(A)*, *dfrG*	rep19b, rep19c, rep20, rep39, rep10,	–	SCC*mec* type I(1B)	I	ST59	ISSep3, ISSau4	3 (0)	0.951 (513)
C127	*mecA*, *blaZ*, *mph(C)*, *msr(A)*, *erm(C)*, *dfrG*	rep10, rep19b, rep19c, rep20, rep39	–	SCC*mec* type I(1B)	I	ST59	ISSau4, ISSep3	4 (0)	0.949 (548)
C133	*blaZ*	None	–	–	–	ST490	ISSep3	4 (0)	0.964 (152)
C135	*mecA*, *blaZ*, *norA*, *tet(K)*, *msr(A)*, *mph(C)*, *cat(pC221)*, *dfrG*	rep7a, repUS70	–	SCC*mec* type IVg(2B)	I	Unknown	ISSep3	3 (0)	0.953 (394)
C137	*mecA*, *norA*, *blaZ*, *aac(6′)-aph(2′′)*, *erm(A)*, *erm(C)*, *dfrG*	rep10, repUS9, repUS43	–	–	–	Unknown	IS256	1 (0)	0.727 (36)
C138	*blaZ*, *dfrG*	–	–	–	–	Unknown	ISSep3, ISSau4	7 (0)	0.955 (328)
C145	*MecA*, *blaZ*, *tet(K)*, *aac(6′)-aph(2′′)*, *aph(3′)-III*, *erm(B)*	rep11a, rep18b, rep2, rep7a	–	SCC*mec* type IVa(2B)	–	ST2	ISSep3, ISSau4, ISEnfa3, IS16, ISEfa5, IS256	3 (0)	0.968 (1)
C146	*MecA*, *blaZ*, *msr(A)*	repUS48	–	SCC*mec* type IVa(2B)	–	ST640	IS30, ISEc36, ISSep2, ISSau4	4 (0)	0.948 (121)
C148	*blaZ*, *tet(K)*	rep21, rep19c, repUS9, rep5d, rep7a	–	–	–	Unknown	IS256	2 (0)	0.948 (30)

R-M system, restriction-modification system; ACME, arginine catabolic mobile element. ACME types I (*arc* and *opp3* operons), II (the *arc* operon only), III (the *opp3* operon only), IV (*arc* and *kdp* operons) and V (*arc*, *opp* and *kdp* operons). Pathogenicity score: prediction of a bacteria’s pathogenicity toward human hosts using PathogenFinder. Strain of the closet pathogenic family linkage: *Staphylococcus epidermidis* ATCC 12228. MRSE, methicillin-resistant Staphylococcus epidermidis; MLST, multilocus sequence typing; CRISPR-Cas, clustered regularly interspaced short palindromic repeats/CRISPR-associated. *SCC*mec* typing was predicted with the SCC*mec*Finder. ^#^Unknown means a sequence type could not be assigned to isolate due to missing alleles in the draft genomes.

Thirteen out of the 16 isolates showed an agreement between the cefoxitin-resistant phenotype and *mecA* gene. The tetracycline-resistant genes *tet(K)* and *tet(M)* were found in 4/5 (80%) of isolates phenotypically resistant to tetracycline. The aminoglycoside resistance mechanisms *aac(6′)-aph(2″)*, *aad*, and *aph(3′)-III* were found in 6/16 (37.5%) of isolates; however, none of those isolates were phenotypically resistant to gentamicin or amikacin. Furthermore, the MLS_B_ resistance mechanisms *msr(A)*, *mph(C)*, *erm(A)*, *erm(B)*, and *erm(C)* were also detected in 11/12 (91.6%) of phenotypically resistant isolates. *dfrG* gene was found in 9/14 (62.3%) of isolates resistant to sulfamethoxazole/trimethoprim. We identified known and putatively novel mutations in *gryA*, *parC*, and *parE* quinolone resistance-determining region (QRDR) genes in some fluoroquinolone-resistant isolates (Table 4). We detected no mutation in the drug target in one of the three isolates found to be resistant to rifampicin. Resistance, in this case, may be mediated by a mechanism yet to be described. We identified the major facilitator superfamily (MFS) antibiotic efflux pump (*norA*), which can also confer resistance to fluoroquinolones. Seven mutations were found in *gyrA* and four mutations in *parC*, but no mutations were detected in *gyrB*. We further found two mutations (S486Y and Y737S) in *rpoB* gene.

**TABLE 4:** Mutations in *gyrA*, *gyrB*, *parC*, and *parE* in the *Staphylococcus epidermidis* isolates.

Isolate ID	*gyrA*	*gyrB*	*parC*	*parE*	*rpoB*
C35	–	–	–		N/A
C135	–	–	*K272R	None	N/A
C137	*V304I	–	*K272R	None	N/A
C40	–	–	*K272R	None	N/A
C38	S84Y, *E888D, *D890E, *S891D, *D892S, *E893D	–	*S80F, *D84Y, *E231K, *K272R	None	N/A
C36^μ^	–	–	*K272R	None	–
C81^μ^	–	–	*K272R	D434V	S486Y
C146^#^	N/A	N/A			*Y737S

Mutations in *gyrA*, *gyrB*, *parC*, and *parE* in the *S. epidermidis* isolates. *Putatively novel mutations. ^#^Isolates resistant to rifampicin only. ^μ^Isolates resistant to both fluoroquinolones and rifampicin.

### Pathogenicity and Virulome

The mean probability of isolates being pathogenic to humans ranged from 0.727 to 0.968 as predicted by PathogenFinder and matched several pathogenic families. The virulome analysis revealed putative virulence genes encoding proteins belonging to multiple virulence categories of *S. epidermidis*, i.e., adherence/biofilm formation, enzymes, immune evasion, secretion, toxin, anti-phagocytosis, intracellular survival, and stress adaptation (Table 5).

**TABLE 5:** Virulence genes identified in MRSE isolates in this study.

Isolate ID	Virulence gene							
	Adherence/biofilm	Enzymes	Immune evasion	Secretion	Toxin	Antiphagocytosis	Intracellular survival	Stress adaptation
C35	*atl*, *ebh*, *ebp*, *sdrE*, *sdrH*, *prgB/asc10*, *dltA*, *ebpC*, *pavA*, *flmH*, *slrA*, *plr/gapA*, *fsrA*, *fsrB*, *fsrC*	*geh*, *lip*, *sspA*, *nuc*, *gelE*, *EF0818*, *stp*, *sprE*	*hasC*, *rfbA-1*, *rmlB*, *rmlD*, *capC*, *manA*	*esaA*, *esaD*, *esaG*, *essA*, *essB*, *essC*, *esxA*	*hlb*	*rmlC*, *cdsA*, *cpsA*, *cpsF*, *gnd*	*lplA1*	*katA*
C36	*atl*, *ebh*, *ebp*, *icaA*, *icaB*, *icaC*, *icaR*	*geh*, *lip*, *nuc*	*adsA*, *capC*, *manA*	–	*hlb*, *cylR2*	–	–	
C38	*atl*, *ebh*, *ebp*, *icaA*, *icaB*, *icaC*, *icaR*, *sdrC*, *sdrG*, *sdrH*, *prgB/asc10*, *dltA*, *ebpC*, *pavA*, *slrA*, *fsrA*, *fsrB*, *fsrC*	*sspB*, *geh*, *lip*, *sspA*, *nuc*, *gelE*, *EF0818*, *stp*, *sprE*	*hasC*, *rfbA-1*, *rmlB*, *rmlD*, *galE*, *manA*	*essC*	*hlb*	*rmlC*, *cdsA*, *cpsA*, *cpsF*, *gnd*	*lplA1*	*katA*
C40	*atl*, *ebh*, *clfA*, *ebp*, *icaA*, *icaB*, *icaC*, *icaR*, *sdrG*, *sdrH*, *prgB/asc10*, *dltA*, *ebpC*, *pavA*, *slrA*, *plr/gapA*	*sspB*, *geh*, *lip*, *sspA*, *nuc*, *gelE*, *EF0818*, *stp*, *sprE*	*hasC*, *rfbA-1*, *rmlB*, *rmlD*, *manA*	*essC*	*hlb*	*rmlC*, *cdsA*, *cpsA*, *cpsF*, *gnd*	*lplA1*	*katA*
C68	*atl*, *ebh*, *ebp*, *sdrH*, *flmH*	*sspB*, *geh*, *lip*, *sspA*, *nuc*	*capB*	–	*hlb*	–	–	
C81	*atl*, *ebh*, *ebp*, *icaA*, *icaB*, *icaC*, *icaR*, *sdrG*, *sdrH*, *asa1*, *dltA*, *ebpC*, *fss3*, *pavA*, *slrA*, *plr/gapA*	*sspB*, *geh*, *lip*, *nuc*, *gelE*, *stp*, *sprE*	*hasC*, *rfbA-1*, *rmlB*, *rmlD*, *galE*, *gtaB*, *manA*	–	*hlb*	*rmlC*, *cdsA*, *uppS*, *gnd*	–	*katA*
C119	*sdrH*	*sspB*, *geh*, *sspA*, *nuc*	–	–	*hlb*		–	
C122	*atl*, *ebh*, *ebp*, *sdrG*, *sdrH*, *hcpB*, *htpB*, *orfH*, *flmH*, *nueA*, *tapT*, *fimC*, *fimD*, *fimD*, *pilU*, *pilQ*, *adeG*, *pgaC*	*sspB*, *geh*, *lip*, *sspA*, *plcN*, *eno*	*galE*, *galU*, *mrsA/glmM*, *pgi*, *acpXL*, *gtaB*	*flgA*, *flgB*, *flgC*, *flgD*, *flgE*, *flgF*, *clpV1*, *yplA*	*hlb*, *hlyA*, *cysC1*	*algU*, *rmlB*, *wbjD/wecB*, *gnd*, *manB*, *uge*, *wzb*, *wzc*	–	*katG*, *katA*, *mntB*, *sodCI*
C127	*atl*, *ebh*, *ebp*, *sdrG*, *sdrH*, *hcpB*, *flmH*, *nueA*, *fimC*, *fimD*, *pilU*, *pilQ*, *pgaC*	*sspB*, *geh*, *lip*, *sspA*, *nuc*, *plcN*, *eno*	*galE*, *galU*, *capC*	*flgA*, *flgE*, *flgF*, *flgG*, *flgJ*, *flgK*, *fliC*, *fliI*, *fliR*, *yplA*	*hlb*	*algU*, *rmlB*, *wbjD/wecB*, *cpsG_1*, *uge*, *wzc*	–	*katG*, *katA*, *mntB*
C133	*atl*, *ebh*, *ebp*, *icaA*, *icaB*, *icaC*, *icaR*, *sdrC*, *sdrH-*	*sspB*, *geh*, *lip*, *nuc*	–	–	*hlb*	–	–	
C135	–	*sspB*, *geh*, *lip*, *sspA*, *nuc*	–	–	*hlb*	–	–	
C137	*ebp*, *icaA*, *icaB*, *sdrF*, *sdrH*, *hcpB*, *htpB*, *orfH*, *flmH*, *nueA*, *tapT*, *fimA*, *fimC*, *fimD*, *pilU*, *pilQ*, *adeG*, *pgaC*	*sspB*, *geh*, *lip*, *plcN*, *eno*	*galE*, *galU*, *mrsA/glmM*, *pgi*, *acpXL*	*flgA*, *flgB*, *flgC*, *flgD*, *flgE*, *flgF*	*hlb*, *hlyA*, *cysC1*	*rmlB*, *wbjD/wecB*, *cpsG_1*, *gnd*, *uge*, *wzc*	–	*katG*
C138	–	–	–	–	–	–	–	
C145	*atl*, *ebh*, *ebp*, *icaA*, *icaB*, *icaC*, *icaR*, *sdrG*, *sdrH*	*sspB*, *geh*, *lip*, *sspA*, *nuc*	–	–	*hlb*	–	–	
C146	*atl*, *ebh*, *ebp*, *sdrG*, *sdrH*, *csgG*, *ecpA*, *fleR*, *fliQ*, *hcpB*, *htpB*, *orfH*, *flgC*, *flgC*, *plr/gapA*, *pilW*, *pgaC*	*sspB*, *geh*, *lip*, *sspA*, *eno*	*galE*, *galU*, *mrsA/glmM*, *pgi*, *acpXL*	*esaA*, *esaD*, *esaE*, *essB*, *essC*, *flgB*, *flgC*, *flgD*, *ipaH*, *clpV*	*hlb*, *hlyA*, *cysC1*	*algU*, *rmlB*, *wbjD/wecB*, *gnd*, *wcaG*, *wcaI*, *wzb*	–	*katG*, *sodCI*
C148	*atl*, *ebp*, *sspB*, *sspC*, *geh*, *sspA*	–	–	–	–		–	

*MRSE, methicillin-resistant *Staphylococcus epidermidis*.*

### Whole-Genome Sequencing-Based Multilocus Sequence Typing

*In silico* MLST analyses identified seven different MLST types, namely, sequence types (ST) ST54 (two), ST83 (one), ST2 (two), ST490 (one), ST640 (one), ST210 (one), and ST59 (two). The most resistant isolate belonged to ST54 and harbored nine ARGs encoding resistance to five antibiotic drug classes (Table 3). The eBURST analyses matched the various STs to the closest global ancestry STs. ST54 matched STs originating from human and animal sources from Denmark, Italy, Japan, India, and Russia. The eBURST analyses also matched ST2 to the highest number of similar clones originating from several countries including Argentina, Cape Verde, Denmark, Germany, Spain, Italy, and Japan (Supplementary Table 1).

### Mobilome and the Genetic Support Environment

*In silico* SCC*mec* typing/subtyping revealed six SCC*mec* types/subtypes: SCC*mec* type IV(2B), SCC*mec* type IVg(2B), SCC*mec* type V(5C2), SCC*mec* type IVa(2B), SCC*mec* type XIII(9A), and SCC*mec* type I(1B). Isolates with SCC*mec* type I(1B) (*n* = 2) belonged to sequence type (ST59), while SCC*mec* type V(5C2) isolates (*n* = 2) belonged to sequence type ST54. The community-acquired SCC*mec* type IV (in various subtypes) was the most common type found. *mecA* gene (but not *mecC*) was detected by SCC*mec*Finder as the sole mechanism of resistance in MRSE isolates. All β-lactam-resistant isolates possessed *blaZ* gene (encoding β-lactamase) and their regulator genes *blaR* and *blaI*.

Plasmid analysis by PlasmidFinder and BacWGSTdb^16^ revealed 24 different plasmid replicon types. Rep10 (10), rep7a (7), and repUS43 (6) were the most predominant plasmid replicon types. Replicon types rep39, repUS9, and rep19b were found in four (25%), four (25%), and five (31.2%) of isolates, respectively.

We found ISs in all 16 isolates. In total, 10 different IS types belonging to six different IS families were detected (Supplementary Figure 1). The most predominant IS families were IS256, 1S200/IS605, and IS3. IS256, closely linked to biofilm formation and virulence in pathogenic MRSE isolates, was found in nine isolates, seven of which had the *ica* operon involved in biofilm formation. The resistance gene *aac(6′)-aph(2″)* was found in association with IS256, while the virulence gene *gelE* (predicted to be linked with *Enterococcus*) was found in association with ISEfa11 in one isolate (C81). Furthermore, the resistance gene *erm(A)* was also found in association with the transposon Tn554 in some isolates. Using NCBI annotation, we found *blaZ* gene surrounded by regulator genes *blaR* and *blaI*. Similarly, *mecA* gene was frequently found with regulatory genes *mecI* (a repressor) and *mecR1* (a sensor inducer) and ISs (IS257 and IS1182). Table 6 describes the genetic support environment of some resistance genes found in this study, with the focus on the association MGEs with the ARGs and virulence genes.

**TABLE 6:** MGEs associated with antibiotic resistance genes in the MRSE strains.

Isolate	Contig	Synteny	Plasmid/chromosomal sequence with closest nucleotide homology (accession number)
C35	237	*msr(A*):recombinase family protein	*Staphylococcus warneri* strain WB224 plasmid pWB224_2 (CP053472.1)
	376	*Tet(K*):TPA	*S. warneri* strain 16A plasmid (CP031268.1)
C36	33	*BlaI*:*BlaR1*:*blaZ*:transposase:tyrosine-type recombinase/integrase	*Staphylococcus epidermidis* strain O47 chromosome (CP040883.1)
	59	*RadC*:*erm(A)*: *ANT(9)-Ia*:transposase: tyrosine-type recombinase/integrase:tyrosine-type recombinase/integrase:*RadC*:recombinase	*Staphylococcus aureus* strain BPH2056 genome assembly, chromosome (LR027874.1)
	81	IS431*mec* (IS257):*mecA*:*MecR1*	*S. aureus* strain Guangzhou-SAU071 chromosome (CP053183.1)
	91	relaxase:*MobC*:*cat(pC233)*	*S. aureus* plasmid pC223 (AY355285.1)
	93	relaxase:*cat(pC221)*:relaxase	*S. aureus* strain 08-028 plasmid (CP045437.1)
C38	42	recombinase:*CcrB*:IS1182:*MecR1*:*mecA*:IS6-like element(IS257)	*S. aureus* strain NZAK3, chromosome (LT009690.1)
	54	*erm(C)*:ErmCL	*S. aureus* strain 18082 chromosome
C40	31	*YycH*:IS6-like element (IS257):*mecA*:*MecR1*	*S. epidermidis* strain HD66 chromosome (CP040868.1)
	61	*Erm(C)*:*ErmCL*:*RepL*	*S. aureus* strain 18082 chromosome (CP041633.1)
C81	30	*BlaI:BlaR1:blaZ*:transposase:*XerC*:Tn554-related transposase A	*S. epidermidis* strain O47 chromosome (CP040883.1)
	144	Recombinase/integrase:Tn554:*ANT(9)-Ia*:*erm(A)*	*S. aureus* strain SR153 chromosome (CP048643.1)
	148	IS6-like element (IS257):*mecA*:*MecR1*	*S. aureus* strain Guangzhou-SAU071 chromosome (CP053183.1)
	160	*cat(pC221*):*Erm(B)*	*Staphylococcus pseudintermedius* strain AH18 chromosome (CP030374.1)
C119	12	recombinase:*BlaI*:*BlaR1*:*blaZ*: type I toxin–antitoxin system	*S. epidermidis* strain SE95 plasmid (CP024439.1)
	204	*Erm(C):*ErmCL	*S. aureus* strain 18082 chromosome (CP041633.1)
C133	6	TPA:Tn554:IS1182:*blaZ*:*BlaR1*:*Bla*I	*S. epidermidis* strain NCCP 16828 chromosome (CP043847.1)
C135	70	resolvase:*CcrB*:IS1182:*MecR1*:*mecA*	*S. aureus* strain ER03750.3 chromosome (CP030557.1)
	75	IS6:*blaZ*:*BlaR1*:*BlaI*:recombinase	*S. epidermidis* strain Z0118SE0260 chromosome (CP060794.1)
	175	*Fst*:*msr(A*):*Mph(C)*	*S. aureus* strain ER02243.3 plasmid (CP030478.1)
C137	31	IS6:*blaZ*:*BlaR1*:*BlaI*:recombinase	*S. epidermidis* strain SESURV_p1_1200 chromosome (CP043796.1)
C138	4	*BlaI*:*BlaR1*:blaZ:TN554:TPA	*S. epidermidis* strain NCCP 16828 chromosome (CP043847.1)
	37	*dfrG*:Insertion element (TPA)	*S. epidermidis* strain NCCP 16828 chromosome (CP043847.1)
C145	30	IS1182:*MecR1*:*mecA*	*S. aureus* strain NRS484 chromosome (CP026066.1)
	34	GNAT family *N*-acetyltransferase:APH(3′)-IIIa:Peptide binding protein:*erm(B)*:antitoxin: peptide-binding protein: IS1216(IS6):recombinase	*Enterococcus faecium* strain VVEswe-R plasmid (CP041269.2)
	36	*Lnu(B)*:*ant(6)-Ia*:sat1):ISEfm1	*E. faecium* strain Efm0123 plasmid (KR066794.1)
	54	GNAT family N-acetyltransferase:*APH(2′′)-Ia*	*Staphylococcus hominis* strain FDAARGOS_661 plasmid (CP054551.1)
C148	17	recombinase:*BlaI*:*BlaR1*:*blaZ*:type I toxin–antitoxin system	*Staphylococcus cohnii* strain FDAARGOS_744 plasmid (CP054810.1)

*MGEs, mobile genetic elements; MRSE, methicillin-resistant *Staphylococcus epidermidis*.*

The PHASTER tool identified intact prophages integrated into the genomes of 10 isolates. PHAGE_Staphy_StB20 (*n* = 9) and PHAGE_Staphy_187 (*n* = 5) were the most predominant prophages. Prophages did not harbor resistance genes. Prophage characteristics, including GC content and number of coding sequences, are shown in Supplementary Table 2.

### Identification and Classification of Clustered Regularly Interspaced Short Palindromic Repeats/CRISPR-Associated Elements, Arginine Catabolic Mobile Element, and Restriction-Modification System

The CRISPRCasFinder identified sequences with CRISPR. All isolates possessed at least one sequence with CRISPR. However, CRISPR-associated (Cas) genes were not detected. Two isolates possessed the R-M system, and both were classified as type II. ACME was identified in five isolates and were classified as type I (three) and type III (two).

### Phylogenetic Relationship Among *Staphylococcus epidermidis* Isolates in This Study and With Other African *Staphylococcus epidermidis* Isolates

**FIGURE 1:**
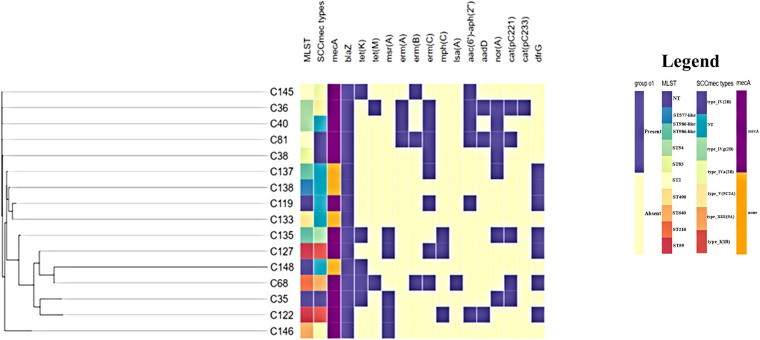
Whole-genome phylogenetic tree annotated with sequence type (ST) assignment, SCC*mec* types, and antibiotic resistance carriage. Gene names above the annotation according to antibiotic class, as follows: β-lactams: *mecA* and *blaZ*; tetracyclines: *tet(K)* and *tet(M)*; macrolide, lincosamide streptogramin B: *msr(A)*, *erm(A)*, *erm(B)*, *erm(C)*, *lsa(A)*, and *mph(C)*; aminoglycosides: *aac(6′)-aph(2″)* and *aadD*; efflux pump: *norA*; trimethoprim: *dfrG*; chloramphenicol: *cat(pC221)* and *cat(pC233)*. The profile was generated using Phandango (https://jameshadfield.github.io/phandango/#/). Heat map at the middle indicates antibiotic resistance gene presence (purple) and absence (yellow). NT, non-typeable.

**FIGURE 2:**
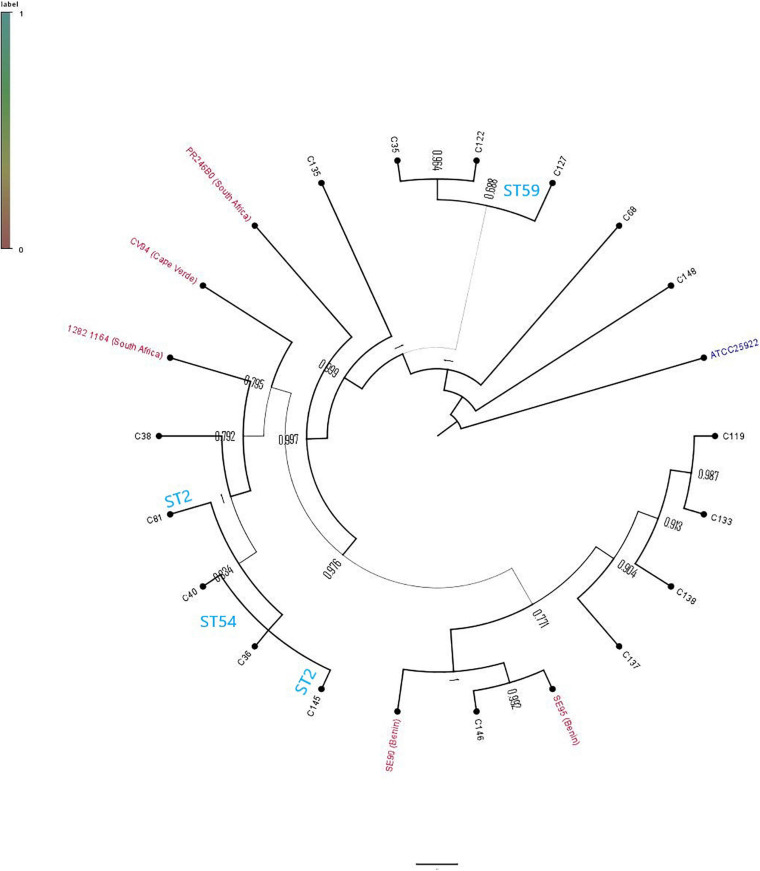
Phylogenetic relationship of *Staphylococcus epidermidis* isolates in this study and African *S. epidermidis* (with their countries indicated) obtained from PATRIC.

The phylogenetic relationship between the 16 study isolates (Figure 1) and five collected isolates from African countries, together with a reference strain, was determined (Figure 2). Generally, the isolates clustered according to STs, with the whole-genome phylogenetics showing higher resolution than the MLST typing scheme. For instance, C145 (ST2) was phylogenetically closer to both C36 and C40 (ST54) than the other ST2 isolate (C81), which was found on different mini branch (Figures 1, 2). However, not much inference could be drawn from the tree analysis due to the small number of deposited *S. epidermidis* genomes from Africa.

## Discussion

In this study, we sequenced MRSE isolates from clinical sources from hospitals in the uMgungundlovu District in the KwaZulu-Natal Province in South Africa. Using WGS, we studied the genomic characteristics, including resistance and virulence determinants, MGEs, and the genetic environments of the resistance genes observed.

Concerning pathogenicity, it has been suggested that there is no apparent genetic difference between commensal non-pathogenic and pathogenic *S. epidermidis* strains, albeit nosocomial *S. epidermidis* strains are boosted with resistance and virulence genes (Méric et al., 2015). The 16 genomes herein analyzed were mostly from pediatric/neonatal patients. *S. epidermidis* is commonly recovered from bacterial bloodstream infections from neonatal units as a probable causative agent (Asante et al., 2020). Children are particularly susceptible to acquiring *S. epidermidis* in perinatal hospitals (Cabrera-Contreras et al., 2019).

We detected various resistance genes encoding resistance to several antibiotic drug classes that explained the observed phenotypic resistance in isolates. Resistance genes found in this study encode enzyme inactivation (β-lactamases), enzyme modification of antibiotic target such as *erm* genes that mediate macrolide–lincosamide–streptogramin B (MLS_B_) resistance, the aminoglycoside-modifying enzymes, replacement of antibiotic target site as in *mecA*-mediated resistance to β-lactams in staphylococci, and MFS antibiotic efflux pump (*norA*), which can also confer resistance to fluoroquinolones (Foster, 2017). There was no association between ARG type and hospital/department. However, *mecA*, *blaZ*, and *norA* genes were found in nearly all isolates from all three hospitals. As well, *erm(C)*, *msr(A)*, *dfrG*, and *mph(C)* genes were distributed across the regional hospital but were not ward specific, while *dfrG*, *tet(K)*, and *erm(B)* were frequently found in the district hospital. The detection of *blaZ* in all isolates and the regulator genes *blaR* and *blaI* may explain the resistance against penicillin and cephalosporins observed in antibiotic susceptibility tests, which is in agreement with a previous study (Cabrera-Contreras et al., 2019). *S. epidermidis* isolates carrying similar resistance gene profiles have been previously reported (Xu et al., 2018; Cabrera-Contreras et al., 2019; Raue et al., 2020).

Generally, there was considerable agreement between resistance phenotypes and genotypes observed with ARGs affirming the phenotype, except in a few instances. The discordance between cefoxitin-resistant phenotype and the *mecA* genotype could be due to alternative mechanisms of resistance, hetero-resistance, or drawback of the present phenotypic testing methods (Band et al., 2019; Harrison et al., 2019).

The lack of phenotypic resistance to gentamicin and amikacin even though aminoglycoside resistance genes were detected could be due the lack of expression of these genes. This could also be due to the fact that amikacin is not affected by most aminoglycoside-modifying enzymes (Doyle et al., 2020). Other factors that can cause discordance between phenotype and genotype include sequence quality of the sample and the read depth of the sequencing platform; insufficient read depth leads to lower coverage (Doyle et al., 2020). The molecular mechanism of resistance in the tigecycline-resistant isolate in this study is unknown, and there was no *tet(X)* gene detected. Similarly, even though resistance phenotypes were observed for teicoplanin, linezolid, and nitrofurantoin, molecular resistance mechanisms were not detected in their genomes. Limitations of current phenotypic detection methods could be responsible for such discrepancies (Cabrera-Contreras et al., 2019). Novel resistance mechanisms may explain these observed phenotypes and are subject to further studies (Osei Sekyere and Asante, 2018).

We found mutations in *gyrA*, *gyrB*, *parC*, *parE*, and *rpoB* genes. In *gyrA* gene, the substitution S84Y, a mutation known to confer fluoroquinolone resistance (Yamada et al., 2008), was detected, while putatively novel mutations V304I, E888D, D890E, S891D, D892S, and E893D were also detected. The effects of the individual mutations on fluoroquinolone resistance were not investigated in this study.

Several virulence genes are shared by both pathogenic and commensal *S. epidermidis* strains (Otto, 2009). Consistent with hospital and commensal *S. epidermidis* strains isolated worldwide (Cabrera-Contreras et al., 2019), our isolates were characterized by adherence/biofilm-forming genes and multidrug resistance. Twelve out of the 16 isolates were biofilm formers as determined by the phenotypic tissue culture plate method, most of which were corroborated by the detection of genes involved in adherence. The *ica* operon and IS256 used as measures of pathogenicity in *S. epidermidis* (Murugesan et al., 2018) were not detected in nine and seven isolates, respectively. This is similar to what was observed in a study in Mexico where 50% of analyzed *S. epidermidis* isolates lacked the *ica* operon (Cabrera-Contreras et al., 2019). The isolates, however, cannot be dismissed as non-pathogenic, as they can deploy several other virulence factors such as immune evasion (encoded by *hasC*, *rfbA-1*, *adsA*, and *capC*), toxins (encoded by *hlb*, *hlyA*, and *cysC1*) anti-phagocytosis (*rmlC*, *cdsA*, *cpsA*, and *cpsF*), stress adaptation (*katA*, *katA*, *mntB*, and *sodCI*), and intracellular survival (*lplA1*). Previous studies in *S. epidermidis* have reported similar biofilm/adherence-associated genes to those reported in this study, including elastin binding protein gene *ebp*, serine protease gene *sspA*, autolysin gene *atlE*, lipase gene *geh*, the cell wall-associated fibronectin-binding protein gene *ebh*, nuclease gene *nuc*, and the *ica* genes (Salgueiro et al., 2017; Xu et al., 2018; Raue et al., 2020).

These antibiotic resistance and virulence genes have been shown to form part of the accessory genome organized within and between species. Prediction of isolates’ pathogenicity toward human hosts yielded a high average probability score (*P*_score_ ≈ 0.937), close to 1.00. This pathogenicity score juxtaposed with the several virulence genes possessed by isolates, supporting their pathogenic potential to humans (Adzitey et al., 2020).

Methicillin-resistant staphylococci have been associated with MGEs, such as the SCC*mec* and ACME. MGEs may be repositories of resistance and virulence genes (Sheppard et al., 2016; Foster, 2017). Their importance is related to their mobile nature, which allows them to transfer from cell to cell, within and between bacterial species through horizontal gene transfer, resulting in frequent exchange of genetic material within the population. The variability observed in the genome of *S. epidermidis* points to the active gene exchange. We thus looked for prophages, CRISPR-Cas system, transposons, and ISs to investigate this phenomenon. The study isolates possessed various ISs that belong to different families. IS256 has been found to be present in pathogenic *S. epidermidis* strains and closely linked with virulence and biofilm formation among MRSE (Murugesan et al., 2018). This observation was affirmed by the fact that all but two of our isolates with IS256 also contained the *ica* operon involved in biofilm formation. Indeed, only one isolate (C133) harbored the *ica* gene that did not have IS256. IS256 has been shown to facilitate genomic rearrangements in pathogenic *S. epidermidis* isolates (Cabrera-Contreras et al., 2019). The detection of IS families IS110, IS200/IS605, and IS3 agrees with a study of Raue et al. (2020), which also found the IS family IS6 was not detected in the current study. Again, the detection of IS256 and the *ica* operon in isolates belonging to ST2 agrees with a study conducted in China, which analyzed *S. epidermidis* from community and hospital environments (Du et al., 2013).

In this study, plasmids rep10, repUS43, rep7a, rep7b, and repUS70 frequently carried resistance genes *erm(C)*, *tet(M)*, *tet(K)*, *catpC233*, and *blaZ*, respectively, whereas other genes were chromosomally mediated. These plasmid-borne genes can easily be transferred by conjugation between cells, spreading resistance (Cabrera-Contreras et al., 2019). Furthermore, although prophages can transfer DNA between cells by transduction, no resistance genes were carried by prophages detected in this study. Transposons detected in the genome of two isolates, like plasmids, may carry genes beneficial to bacteria, such as those involved in antibiotic resistance (Mbelle et al., 2019). They can transpose from the chromosome and can move to different sites of the DNA within a cell. In this study, the transposon Tn554 was found to flank the resistance gene *erm(A)* in some isolates, which may allow it to jump between the chromosome and the plasmid. The small sample size of isolates in this study limits associations between ARGs, virulence genes, MGEs, and isolate demographics. However, the carriage of ARGs on diverse MGEs enhances the mobilization and dissemination of these genes.

CRISPR-Cas system is a defense mechanism deployed by bacteria against phage infection. After surviving a viral infection, certain bacteria imprint a piece of the viral genetic code as a memory of the infection. Bacteria may use this to neutralize future infections caused by similar viruses by cleaving the viral genetic sequence before they can take control of the bacterial host (Makarova et al., 2020). All isolates bore at least one sequence with CRISPR (Table 3), 10 of which contained at least one intact prophage. In a previous study (Raue et al., 2020), four candidates for CRISPR elements were found while investigating the genome of *S. epidermidis* O47 strain, which is biofilm-positive and methicillin-susceptible. The *S. epidermidis* O47 strain from that study lacked the CRISPR-associated genes just, as was observed in this study. The two isolates found with the R-M system were classified as type II. Like the CRISPR-Cas system, the R-M system is a defense system developed by bacteria against invasion by bacteriophages (Vasu and Nagaraja, 2013).

The ACME system, a pathogenicity island, has generated interest and is thought to facilitate the host colonization and immune evasion and to transport virulence or survival genes (O’Connor et al., 2018). ACME elements were detected in 31.3% of study isolates. This is lower than the prevalence of 40% to 65.4% reported in MRSE in a study that investigated the diversity of the ACME in *S. epidermidis* from the oral cavity and periodontal pockets (O’Connor et al., 2018). In comparison, an ACME carriage of 16% was detected in a study that compared the resistance and virulence profile of *S. epidermidis* isolates from bloodstream infections and nares of neonates (Salgueiro et al., 2017). ACME shows a higher prevalence and greater diversity in *S. epidermidis* compared with *S. aureus*. In *S. aureus*, studies have proved that ACME is usually incorporated in bacterial chromosome adjoining the SCC*mec*IV element (Diep et al., 2006; Ellington et al., 2008). In this study, however, no association between ACME and SCC*mec* type IV was found, which is consistent with results obtained by Du et al. (2013). Most of the resistance genes were bracketed by either transposases or ISs or a combination of both, and these can transfer resistance genes within and between plasmids and chromosomes (Mbelle et al., 2019) potentially within and between bacterial species.

*cat(pC233)* gene was bracketed by plasmid mobilization relaxosome protein MobC in isolate C36, while *erm(C)* gene was bracketed by the 23S rRNA methylase leader peptide ErmCL and replication/maintenance protein RepL. In two isolates (C148 and C119), *blaZ* gene was surrounded by the type I toxin–antitoxin system, which may play a role in biofilm and persister cell formation (Wang and Wood, 2011; Table 6 and Figure 1).

Multilocus sequence typing has shown the population structure of *S. epidermidis* to be clonal (Thomas et al., 2007). The clonal lineages ST2, ST5, and ST23, which are the most commonly reported in hospital environments as well as other sequence types of *S. epidermidis* are globally distributed (Miragaia, 2018). ST2, in particular, is predominant in the hospital environment. In this study, 2/16 isolates belonged to ST2. Both ST2 isolates in this study possessed *icaA* gene and IS256, both of which are linked to enhanced pathogenicity (Du et al., 2013). ST35, ST81, and ST89 were not represented in this study, consistent with global data (Cabrera-Contreras et al., 2019). Despite the relatedness of isolates, there is still considerable variation within individual isolates pointing to their mobilization on diverse MGEs.

## Conclusion

*Staphylococcus epidermidis* isolates from public hospitals in uMgungundlovu exhibited several permutations and combinations of ARGs, virulence genes, and MGEs pointing to a complex milieu of mobilized antibiotic resistance and pathogenic characteristics in clonal and multiclonal strains. The study thus reiterates the need for the genomic surveillance of CoNS as emerging pathogens to gain insights into their potential pathogenicity.

## Data Availability Statement

The datasets presented in this study can be found in online repositories. The names of the repository/repositories and accession number(s) can be found in the article/Supplementary Material.

## Ethics Statement

Ethical approval for the study was obtained from the Biomedical Research Ethics Committee of the University of KwaZulu-Natal under reference number BREC/00001302/2020. This study was a sub-study of the overarching research program on Antibiotic Resistance and One Health (Reference: 473 BCA444/16).

## Author Contributions

JA, DA, AA, and SE conceptualized the work. JA carried out the experiments, analyzed the data, and drafted the manuscript. DA and AA analyzed the data and undertook critical revision of the manuscript. BH, LB, and SE undertook critical revision of the manuscript. All authors have contributed to the final version.

## Conflict of Interest

SE is the chairperson of the Global Respiratory Infection Partnership and member of the Global Hygiene Council, both sponsored by unrestricted educational grants from Reckitt and Benckiser (Pty.) UK. The remaining authors declare that the research was conducted in the absence of any commercial or financial relationships that could be construed as a potential conflict of interest.

## Publisher’s Note

All claims expressed in this article are solely those of the authors and do not necessarily represent those of their affiliated organizations, or those of the publisher, the editors and the reviewers. Any product that may be evaluated in this article, or claim that may be made by its manufacturer, is not guaranteed or endorsed by the publisher.

## Footnotes

1
https://github.com/najoshi/sickle

2
https://pathogen.watch

3
https://card.mcmaster.ca/analyze/rgi

4
https://cge.cbs.dtu.dk/services/VirulenceFinder/

5
http://www.mgc.ac.cn/cgi-bin/VFs/v5/main.cgi?func=VFanalyzer

6
http://bacdb.cn/BacWGSTdb

7
https://cge.cbs.dtu.dk/services/MLST/

8
https://pubmlst.org/

9
https://cge.cbs.dtu.dk/services/SCCmecFinder/

10
https://phaster.ca/

11
https://cge.cbs.dtu.dk/services/MobileElementFinder/

12
https://cge.cbs.dtu.dk/services/

13
https://www.patricbrc.org/

14
https://cge.cbs.dtu.dk/services/CSIPhylogeny/

15
http://tree.bio.ed.ac.uk/software/figtree/

16
http://bacdb.org/BacWGSTdb/
