# Supplementary material for: Genomic Analysis of Antibiotic-Resistant Staphylococcus epidermidis Isolates From Clinical Sources in the Kwazulu-Natal Province, South Africa
Source: Front Microbiol. 2021 Aug 5;12:656306. doi: 10.3389/fmicb.2021.656306 (PMC8374169; doi:10.3389/fmicb.2021.656306)
Supplement: Supplementary file 1 [file Data_Sheet_1.docx]

**Table S1.** A table showing the eBURST (Based Upon Related Sequence Types) analyses of the study sequence types with global curated STs in PubMLST database.

| MLST (Isolate) | Type of clone | Closet global ancestry sequence type (ST) | Source |
| --- | --- | --- | --- |
| ST54 (C36, C40) | Similar ^a^ | ST54 | Human, Animal |
| ST83 (C38) | Similar ^a^ | ST83 | Human |
| ST210 (C68) | Similar ^a^ | ST210 | Human |
| ST2 (C81, C145) | Similar ^a^ | ST2 | Human, Environment |
| ST59 (C122, 127) | Similar ^a^ | ST59 | Human, Animal |
| ST490 (C133) | Similar ^a^ | ST490 | Human |
| ST640 (C146) | Similar ^a^ | ST640 | Human |

Table S2 Distribution of intact prophage regions among the MRSE strains

| **Sample code** | **Intact prophages** | **Region** | **Length (kb)** | **No.CDS** | **GC%** | **Phage** | **Resistance genes** |
| --- | --- | --- | --- | --- | --- | --- | --- |
| C35 | - | - | - | - | - | - | - |
| C36 | 2 | 1 | 34 |  | 28.44 | PHAGE_Staphy_187 | - |
|  |  | 2 | 30.8 | 30826 | 33.42 | PHAGE_Staphy_StB20_like | - |
| C38 | 2 | 1 | 34 |  | 28.43 | PHAGE_Staphy_187 | - |
|  |  | 2 | 65 |  | 32.95 | PHAGE_Staphy_StB20_like | - |
| C40 | 2 | 1 | 34 | 34053 | 28.44 | PHAGE_Staphy_187 | - |
|  |  | 2 | 41.6 | 41681 | 32.7 | PHAGE_Staphy_StB20_like | - |
| C68 | - |  |  |  |  |  | - |
| C81 | 3 | 3 | 30.4 | 30449 | 28.63 | PHAGE_Staphy_187 | - |
|  |  | 4 | 24.3 | 24394 | 34.24 | PHAGE_Staphy_StB20 | - |
|  |  | 5 | 21.6 | 21691 | 33.7 | PHAGE_Staphy_StB20 | - |
| C119 | 1 | 1 | 34.1 | 34102 | 36.47 | PHAGE_Entero_vB_IME197 | - |
| C122 | 1 | 1 | 42.7 | 42796 | 34.14 | PHAGE_Staphy_StB12 | - |
| C127 | - | - | - | - | - | - | - |
| C133 | 1 | 1 | 50.5 | 50527 | 34.29 | PHAGE_Staphy_StB12 | - |
| C135 | - | - | - | - | - | - | - |
| C137 | 3 | 1 | 44.8 | 44882 | 54.65 | PHAGE_Salmon_SEN34 | - |
|  |  | 3 | 17.5 | 17545 | 55.52 | PHAGE_Klebsi_phiKO2 | - |
|  |  | 5 | 40.9 | 40943 | 55.52 | PHAGE_Staphy_StB20 | - |
| C138 | 1 | 2 | 40.9 | 40943 | 33.98 | PHAGE_Staphy_StB20 | - |
| C145 | 3 | 1 | 52.2 | 52234 | 29.21 | PHAGE_Staphy_187 | - |
|  |  | 2 | 41 | 41071 | 32.54 | PHAGE_Staphy_StB20_like | - |
|  |  | 4 | 35.6 | 35674 | 36.21 | PHAGE_Entero_vB_IME197 | - |
| C146 | - | - | - | - | - | - | - |
| C148 | - | - | - | - | - | - | - |

Figure S1: A bar chart depicting the total number of each predicted insertion sequence (IS) families in the MRSE isolates
